# Avian tail ontogeny, pygostyle formation, and interpretation of juvenile Mesozoic specimens

**DOI:** 10.1038/s41598-018-27336-x

**Published:** 2018-06-13

**Authors:** Dana J. Rashid, Kevin Surya, Luis M. Chiappe, Nathan Carroll, Kimball L. Garrett, Bino Varghese, Alida Bailleul, Jingmai K. O’Connor, Susan C. Chapman, John R. Horner

**Affiliations:** 1 Department of Cell Biology and Neuroscience, Montana State University, Bozeman, MT 59717 USA; 2 Honors College, Montana State University, Bozeman, MT 59717 USA; 3 Dinosaur Institute, Los Angeles County Museum of Natural History, Los Angeles, CA 90007 USA; 4 Section of Ornithology, Los Angeles County Museum of Natural History, Los Angeles, CA 90007 USA; 5 Keck School of Medicine, University of Southern California, Los Angeles, CA 90033 USA; 6 Department of Pathology and Anatomy, University of Missouri, Columbia, MO 65211 USA; 7 Key Laboratory of Vertebrate Evolution and Human Origins, Institute of Vertebrate Paleontology and Paleoanthropology, Chinese Academy of Sciences, Beijing, 10010 China; 8 Department of Biological Sciences, Clemson University, Clemson, SC 29634 USA; 9 Chapman University, Orange, CA 92866 USA

## Abstract

The avian tail played a critical role in the evolutionary transition from long- to short-tailed birds, yet its ontogeny in extant birds has largely been ignored. This deficit has hampered efforts to effectively identify intermediate species during the Mesozoic transition to short tails. Here we show that fusion of distal vertebrae into the pygostyle structure does not occur in extant birds until near skeletal maturity, and mineralization of vertebral processes also occurs long after hatching. Evidence for post-hatching pygostyle formation is also demonstrated in two Cretaceous specimens, a juvenile enantiornithine and a subadult basal ornithuromorph. These findings call for reinterpretations of *Zhongornis haoae*, a Cretaceous bird hypothesized to be an intermediate in the long- to short-tailed bird transition, and of the recently discovered coelurosaur tail embedded in amber. *Zhongornis*, as a juvenile, may not yet have formed a pygostyle, and the amber-embedded tail specimen is reinterpreted as possibly avian. Analyses of relative pygostyle lengths in extant and Cretaceous birds suggests the number of vertebrae incorporated into the pygostyle has varied considerably, further complicating the interpretation of potential transitional species. In addition, this analysis of avian tail development reveals the generation and loss of intervertebral discs in the pygostyle, vertebral bodies derived from different kinds of cartilage, and alternative modes of caudal vertebral process morphogenesis in birds. These findings demonstrate that avian tail ontogeny is a crucial parameter specifically for the interpretation of Mesozoic specimens, and generally for insights into vertebrae formation.

## Introduction

As increasing numbers of Mesozoic avian fossils are discovered, analyses of these specimens are continually modifying our views of bird evolution. The biology of extant birds provides a framework for interpreting the fossil specimens, and while extensive research has been conducted on multiple and varied aspects of extant birds, many features remain to be studied. One such feature that is often ignored but paradoxically is critical to avian evolution is the tail. The tail underwent considerable morphological change, from the long, reptilian-like ancestral condition to the short, distally fused tail of pygostylian birds. While the search for intermediate species in the transition to short tails has been extensive, the consequences of ontogeny have not been sufficiently considered. This investigation of avian tail ontogeny reveals several phenomena critical not only to interpretation of Mesozoic specimens, but also to the study of vertebral morphogenesis.

The transition from long to short tails coincided with the emergence of the Pygostylia group, which includes those birds with the compound skeletal pygostyle structure at the distal end of a shortened tail^1,2^. Additional changes in the avian axial skeleton, including substantial fusion of sacral vertebrae and bone fusions in the extremities have persisted in modern birds, and are a testament to their adaptive advantages. The contributions of these bone fusions and tail truncation to a more streamlined, fortified skeleton are thought to improve flight dynamics and stability^3^. As such, the transition to short tails played a substantial role in bird evolution and the eventual domination of volant-derived ecological niches.

Numerous fossil discoveries from the Late Jurassic and Early Cretaceous are filling in the gaps of the long- to short-tailed transition. *Archaeopteryx*, a Jurassic dinosaur generally recognized as the first bird, exhibited a mosaic suite of features both avian- and non-avian-like^4–6^. Its designation as avian marks the somewhat contested emergence of true flight, but the increasing discoveries of additional paravians blurs that line. The evolution of birds was clearly a continuum, and had as its foundation a myriad of feathered, flapping maniraptoran dinosaurs. Among the Cretaceous paravians were *Jeholornis* and *Microraptor*. These, like *Archaeopteryx*, sported long ancestral tails and a lack of a distal pygostyle. Interestingly, they coexisted with pygostylian birds, as evidenced by their colocalization in the Jehol fossil beds in Northeastern China^7,8^.

Earlier analyses of vertebrate mutations indicated that at least some pygostylian features could have arisen from very few mutations. These analyses were conducted in mice, and indicated that mutations that cause tail vertebrae to fuse also usually decrease the number of caudal vertebrae^9^. Additional pleiotropic effects of tail shortening and tail fusing mutations include more extensive vertebral fusion along the axial column, as well as peripheral fusions in the limbs, akin to the bone fusions observed in Pygostylia birds^9^. Applying these mutation effects to the fossil record, they point to a distinct possibility that the abrupt transition to short tails could have been the result of multiple pleiotropic effects of a relatively few number of Cretaceous mutations. In addition, they suggest that certain intermediate species in the long- to short-tailed avian transition were less likely to have occurred, such as species with short tails lacking a pygostyle^10^. Strong selection for truncated tails likely also contributed to the relatively sudden appearance of short-tailed birds in the fossil record. A shortened tail is advantageous for flight, as evidenced by reduced tails in other flying vertebrates such as pterosaurs and bats, and by analyses indicating shorter tails are more aerodynamically favorable^11^. The tail of *Pteranodon*, a short-tailed pterodactyloid, has been described as distally fused^12^, reinforcing a potential link between tail shortening and caudal vertebral fusion. *Caudipteryx*, a non-avian oviraptorosaur, also had a distally fused tail with fewer caudal vertebrae than other oviraptorosaurs lacking distal fusion^13^. More discoveries of Cretaceous avians will help resolve the question of intermediates, either by identifications of transitional species, or by the continued lack of them.

The absence of information on early ontogenetic changes in the extant bird tail, and the consequential inability to extrapolate that information to juvenile Cretaceous specimens prompted this study. Pygostyle formation was analyzed across a variety of extant bird clades, and was found to occur as a progressive post-hatching event. Evidence for post-hatch pygostyle fusion was also found in Mesozoic specimens, presented in this study and elsewhere in the literature. Additional analyses revealed three previously undescribed phenomena in extant birds: 1), mineralized caudal vertebral shape changes considerably post hatching; 2), caudal vertebrae are derived from different forms of cartilage; and 3), vertebral process morphogenesis can be achieved by alternate mechanisms. The number of pygostyle vertebrae was also examined, and it was found that some groups of Cretaceous birds incorporated significantly more vertebrae into their pygostyle than other groups of extant and extinct avians. Relevant to Mesozoic specimens, the lack of a pygostyle in juveniles and a variable number of relatively featureless caudal vertebrae can blur the distinction of transitional species. These data call for a reinterpretation of specimens such as *Zhongornis*^14^, a juvenile proposed to be an intermediate in the long- to short-tailed avian transition, and the recent discovery of the coelurosaur tail embedded in amber^15^. Consideration of ontogeny events is therefore crucial to the interpretation of Cretaceous juvenile specimens.

## Results and Discussion

### Chicken pygostyle formation

**Figure 1:**
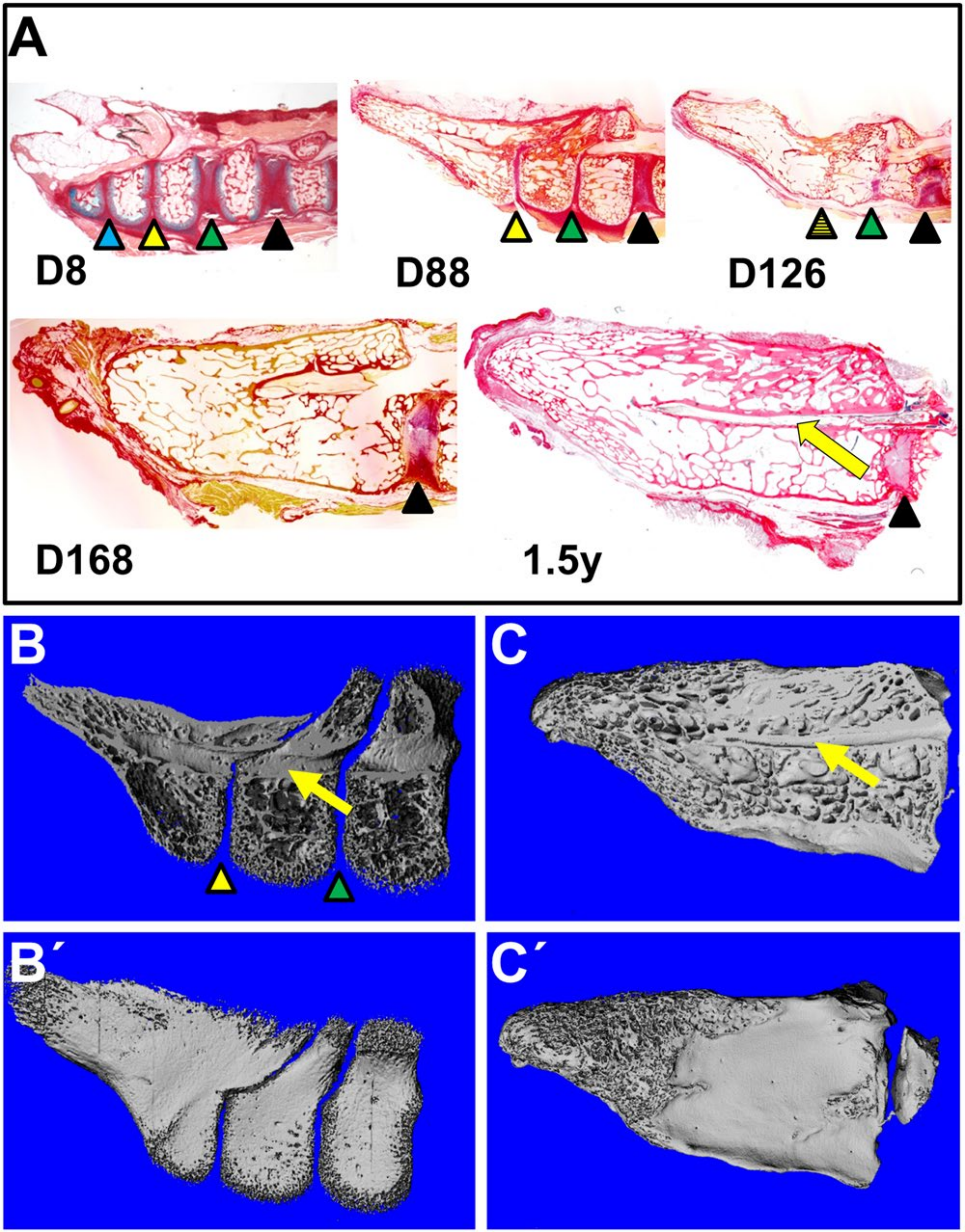
Timeline of pygostyle formation in the chicken. (**A**) Alcian blue and picrosirius red histology staining on semi-thin paraffin sections of progressively older pygostyles, mid-sagittal views, distal to the left and dorsal to the top. At 8 days post hatching, D8, intervertebral discs are present and no fusion is observed, but by D168, all ossified pygostyle vertebrae have fused. Intervertebral discs are marked with color-coded arrowheads to track their progressive dismantling by tissue remodeling; the hatched arrowhead indicates a disc in the process of disassemblage during vertebral fusion. The yellow arrow in this and subsequent panels indicates the spinal cord channel. Fusion of chicken pygostyle vertebrae therefore requires 5 months for completion, and occurs in the distal to proximal direction. (**B**,**B′**) MicroCT scan of 7–8 week old juvenile chicken pygostyle, mid-sagittal and surface views, respectively; distal to the left. MicroCT scanning confirms unfused pygostyle vertebrae in the juvenile. (**C**,**C**′) MicroCT scan of 1.5 year old adult chicken pygostyle, mid-sagittal and surface views, respectively. After pygostyle vertebrae fusion, the spinal cord channel is retained but trabecular bone remodeling removes all traces of intervertebral discs.

The chicken was utilized as an easily accessible model to study pygostyle formation, which had not been previously investigated in depth. Successively aged chicken tails from late embryonic stages to six months post hatching were collected and analyzed by histology (Fig. 1A). While some studies have suggested that pygostyle fusion (and hence formation) occurs during embryonic cartilage development^9,16,17^, closer examination shows that pygostyle vertebrae in the chicken do not fuse until post hatching. Discrepancies with previous studies are likely due to differences in how the analyses were conducted, largely by wholemount alcian blue and alizarin red staining. Alcian blue stains different types of cartilage equivalently, so that both intervertebral discs and cartilaginous pre-vertebrae were indistinguishable, making the pygostyle region in embryo stages appear as a single fused structure. Alcian blue with picrosirius red on semi-thin paraffin sections, however, provides enhanced resolution and enables differentiation between the red cartilage of the intervertebral from the blue cartilage of the vertebral endplates. To verify unfused mineralized pygostyle vertebrae after hatching, juvenile chicken pygostyles were further analyzed by microCT scanning (Fig. 1B,C), which confirmed separate pygostyle vertebrae at seven to eight weeks old. In the chicken, pygostyle fusion is progressive, and occurs in the distal to proximal direction. At eight days post-hatching, all four core pygostyle vertebrae are separate and distinct. By five months of age, the core pygostyle vertebrae are fully fused. Pygostyle formation in the chicken, therefore, occurs as a post-hatch event between ossified vertebrae.

Internal pygostyle anatomy in extant birds has not been previously described, and several interesting features of the fusion process itself are observed. Intervertebral discs, for example, initially form but are subsequently lost by tissue remodeling, though the discs within the pygostyle are narrower than in the free caudal vertebrae (the unfused vertebrae proximal to the pygostyle) (Fig. 1A). The pygostyle discs may be an atavistic trait, indicative of a long-tailed ancestor. In addition, the spinal cord persists in the pygostyle after fusion, and terminates prior to the distal end in the chicken (Fig. 1B,C). Persistence of the spinal cord channel was also observed in a *Confuciusornis* pygostyle^18^, indicating this aspect has been conserved across bird evolution. By 1.5 years of age in the chicken, complete trabecular bone remodeling within the pygostyle erases any trace of intervertebral discs, and hence any trace of the original separate vertebrae (Fig. 1C).

Pygostyle formation occurring after vertebral ossification is highly relevant to the interpretation of juvenile Cretaceous specimens, particularly at the long- to short-tailed transition. The fossil record is devoid of clear transitional forms, which could be due to rapid cladogenesis, in which species with transitional morphologies are less likely to be recovered. The search for potential intermediate species has been extensive, and one possible candidate was identified. *Zhongornis haoae*, from the Yixian Jehol Biota, dating to 125 Ma, was postulated to be a short-tailed bird lacking a pygostyle^14^. This specimen, however, is a juvenile, and its pygostyle may not yet have fused in ontogeny. Until a full-grown specimen is discovered, to indicate whether adults of this species had unfused pygostyle vertebrae, the *Zhongornis haoae* holotype cannot be definitively identified as an intermediate in the long- to short-tailed transition. *Zhongornis* thus exemplifies the difficulties in interpreting the caudal morphology of juvenile specimens.

### Juvenile vertebral shape differs significantly from adults

Ossification sequence is a critical parameter to consider when analyzing juvenile fossils. The vertebral ossification sequence has best been described in humans, and begins in the embryo with formation of three primary ossification centers, one within the primary cartilage model of the vertebral centrum and two for the two halves of the neural arch cartilage model^19^. The vertebral centra, followed by the medial components of the neural arches, ossify by endochondral ossification. Ossification of the distal ends of the spinous and transverse processes, however, does not begin until puberty (as evidenced by x-ray scans, described as secondary ossification centers), and is not completed until approximately 25 years of age^20^. In humans, therefore, the ultimate shapes of bony vertebrae are not established until early adulthood.

**Figure 2:**
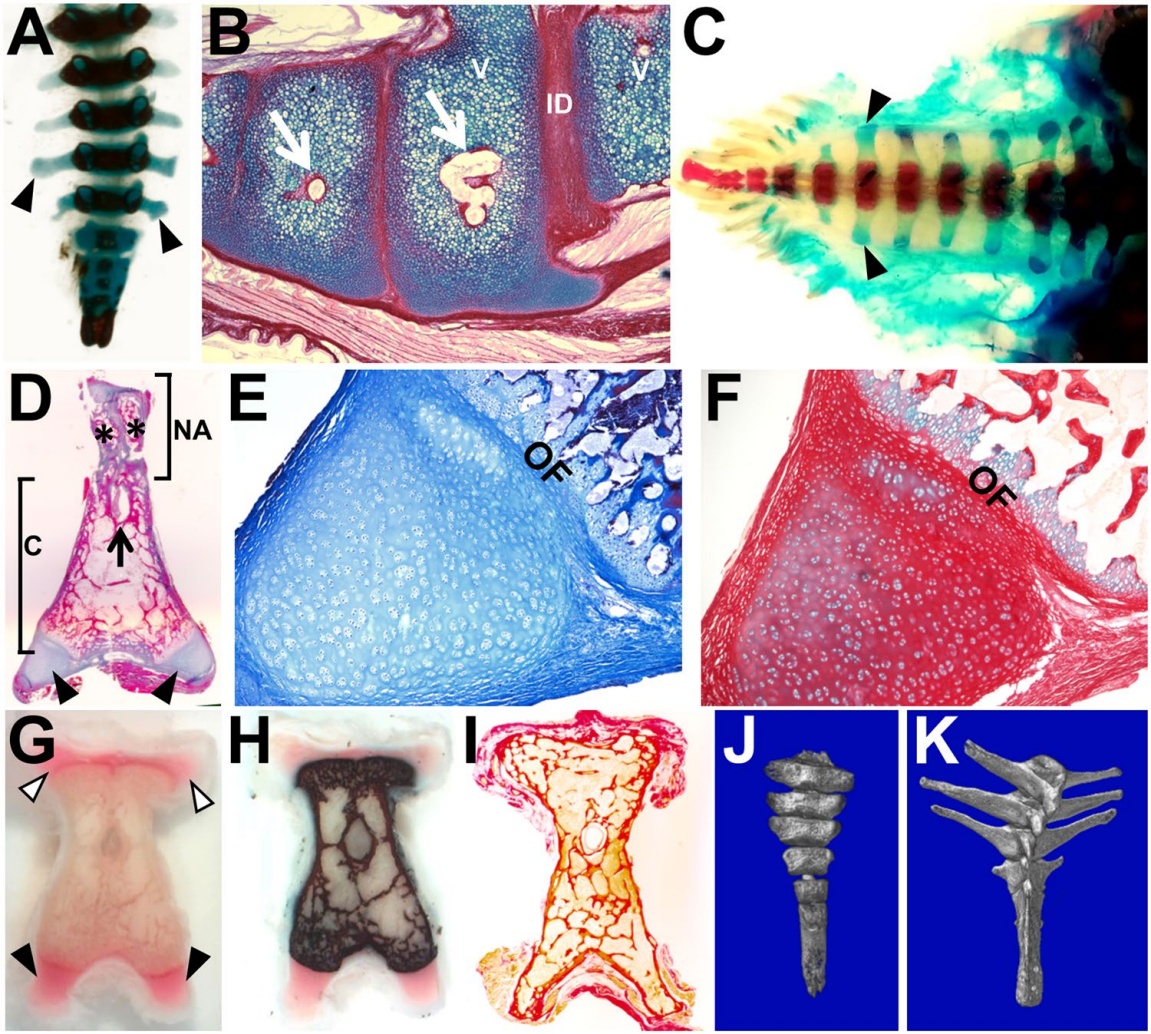
Mineralized shapes of tail vertebrae change significantly during ontogeny. (**A**) Chicken embryonic day E19 wholemount alcian blue and alizarin red; dorsal view, proximal to the top. Alcian blue stains unmineralized cartilage blue and alizarin red stains mineralized tissue red (appears black in this image). In all panels where present, black arrowheads indicate parapophyses. (**B**) Chicken E19, sagittal view, ossification centers in the pygostyle region; alcian blue and picrosirius red; distal to the left; white arrows indicate ossification centers. (**C**) Chicken D4, wholemount alcian blue and alizarin red; dorsal view; distal to the left. (**D**) Chicken 7–8 week old juvenile, pygostyle transverse section, Masson’s Trichrome; dorsal to the top, ventral to the bottom; black arrow indicates spinal cord channel; asterisks indicate unfused medial arch halves. (**E)** Higher magnification of left parapophysis from (**D**). (**F**) Chicken 7–8 week old juvenile, transverse section of left parapophysis, alcian blue and picrosirius red. Compare the red, more sparsely cellular cartilage in this parapophysis tissue with the blue hyaline cartilage in (**B**) (same stain), indicating disparate matrix staining likely due to increased collagen in the parapophysis. (**G**) Chicken 7–8 week juvenile, pygostyle transverse fresh cut section, unstained; white arrowheads indicate transverse process extensions. (**H**) Same tissue as in (**G**) von Kossa, which stains mineralized tissue black. (**I**) Chicken 4+ years old, pygostyle transverse section, alcian blue and picrosirius red. Note that trabecular bone remodeling has extended to the tips of transverse processes and parapophyses. (**J**) MicroCT scan, juvenile *Eurypyga helias* (LACM 104451) partial tail with pygostyle, dorsal view. (**K**) MicroCT scan, adult *E. helias* (LACM 90009) partial tail with pygostyle. Note the mineralized extensions of parapophyses in the adult relative to the juvenile tail vertebrae. Abbreviations: c - centrum, ID - intervertebral disc; NA - neural arch; OF - ossification front; V - vertebra. LACM is the abbreviation for the Los Angeles County Museum of Natural History, followed by the specimen number.

Caudal vertebral ossification in the chicken was followed to determine whether ontogenetic change of calcified vertebral shape is also observed in birds (Fig. 2). For this study, a transverse process is defined as a lateral process derived from the neural arch^21^. Transverse processes are observed as dorsolateral projections in free caudal and pygostyle vertebrae (Fig. 2G, white arrowheads). The more substantial vertebral processes emanate ventrolaterally from the centrum. These have previously been described as transverse processes^22^, but because they are not neural arch-derived, are termed here as parapophyses (Fig. 2C,G black arrowheads). At embryonic day 19 (E19), approximately two days prior to hatching, wholemount alcian blue and alizarin red staining shows that primary ossification centers have formed to the end of the tail, but all tail parapophyses remain cartilaginous and uncalcified (Fig. 2A). Endochondral ossification of vertebral centra in the pygostyle region is observed at this stage (Fig. 2B). By four days post hatching, the centra have ossified, but parapophyses have still not calcified throughout the entire tail (Fig. 2C). At the seven to eight week juvenile stage, von Kossa and Masson’s trichrome staining (Fig. 2D,H) reveal that in the (partially fused) pygostyle, calcification of centra and medial neural arch components has occurred, but notably, the distal ends from both the transverse processes and parapophyses remain cartilaginous and uncalcified. Humans and birds, therefore, share an ontogenetic pattern whereby the tips of vertebral processes do not ossify until later in ontogeny.

Interestingly, histology stains at the 7 to 8 week stage indicate that the cartilages of the transverse and parapophyses tips are morphologically identical, but are not equivalent to the hyaline primary cartilage model of the embryonic vertebral centra, with significantly greater deposition of fibrous matrix, likely collagen (compare Fig. 2B with Fig. 2F). These cartilages, while fiber-rich, are not to be confused with the fibrocartilage of the ligaments that attach to the tips. Notably, the ossification front between the centrum and the tips presents as an epiphyseal plate, with the distal cartilage serving as the zone of reserve or resting cartilage. With this arrangement, growth occurs in the outward direction only, allowing for elongation of ossified processes during this later-stage event. Caudal vertebral bodies, therefore, are formed from different forms of cartilage that remodel to bone at distinct stages.

**Figure 3:**
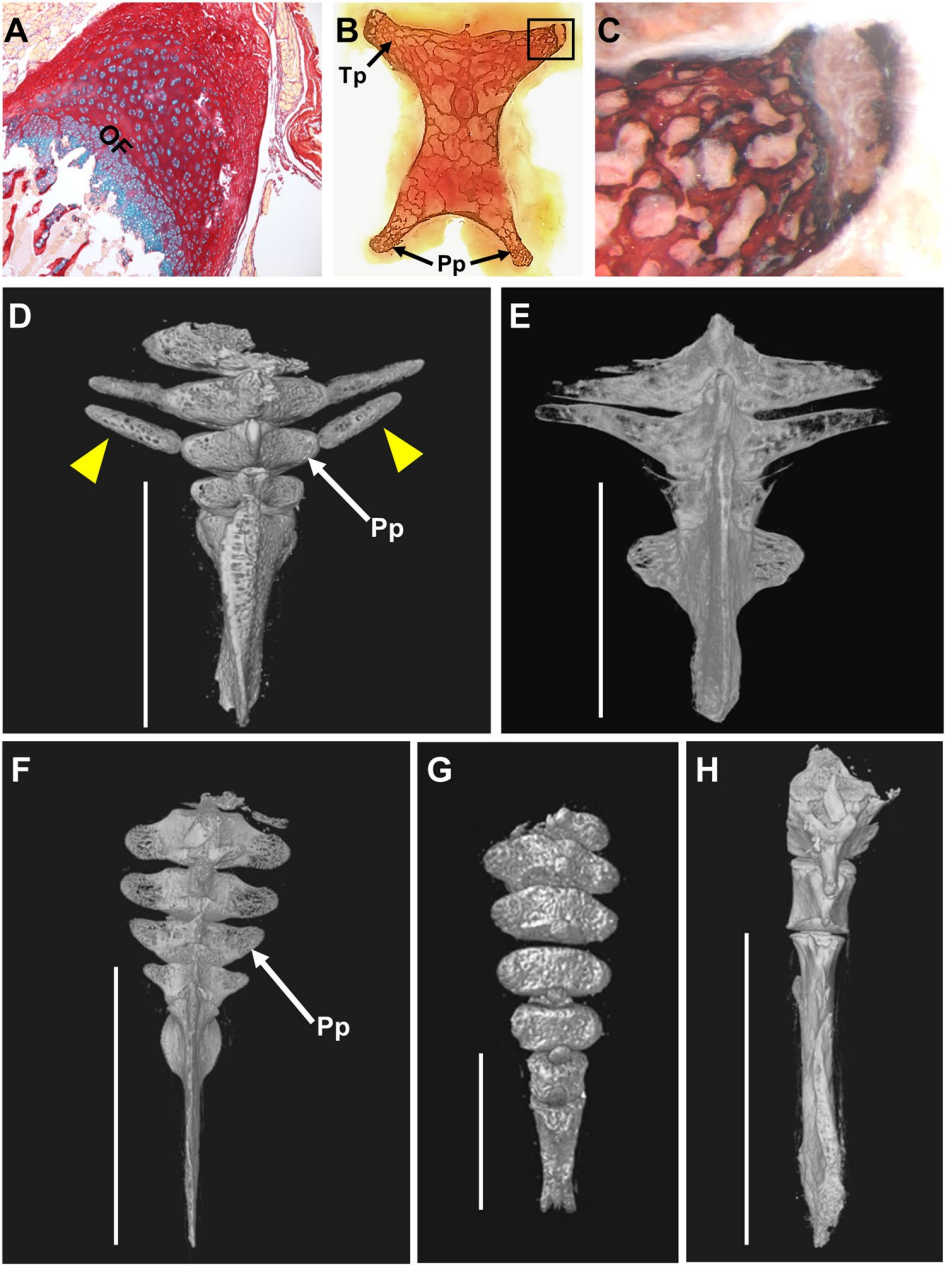
Commonalities and differences in avian caudal vertebral morphogenesis. (**A–F**) Morphogenesis of parapophyses (Pp) and transverse processes (Tp) occurs by epiphyseal-plate-mediated ossification, but further extension can be achieved by fusion of additional ossified elements. (**A**) 4.5 month old emu transverse process from a free caudal vertebra, transverse paraffin section, stained with alcian blue and picrosirius red. The morphology of distal transverse process cartilage in the emu is indistinguishable from equivalent staining in chicken parapophyses (see Fig. 2F), but these cartilages differ from the embryonic hyaline cartilage model (Fig. 2B); (**B**) chicken D126 proximal pygostyle transverse section, von Kossa stained, area in black rectangle magnified in (**C**) showing nearly complete extension of ossified transverse process by epiphyseal plate-mediated ossification that progressed in the medial to distal direction. (**D**) Juvenile lesser nighthawk (LACM 111218) microCT dorsal surface view, showing separate ossified elements (yellow arrowheads), analogous to ribs, that fuse into the parapophyses (white arrow) of free caudal vertebrae (pygostyle region noted by white bar). Complete fusion of these bony elements to vertebral bodies can be seen in (**E**) a microCT dorsal view of an adult lesser nighthawk (LACM 73857). (**F–H**) MicroCT dorsal views of juvenile tails with no evidence of caudal ribs; (**F**) juvenile red-headed woodpecker (LACM 111600), this specimen is at a similar ontogenetic stage, but shows no evidence of the rib-like bony elements. Caudal ribs are also absent in (**G**) a juvenile western screech-owl (LACM 111461) and in (**H**) a developmentally more advanced elegant tern (LACM 116139).

While extension of transverse processes in the emu appears to employ the same mechanism (Fig. 3A), evidence for another strategy can be seen in the lesser nighthawk, *Chordeiles acutipennis*. The juvenile nighthawk appears to have additional separate ossified bones, akin to ribs, that fuse into the parapophyses (Fig. 3D,E). Caudal ribs have been documented in extinct reptiles, in Phytosauria^23^, Icthyosauria^24^, and Plesiosauria^25^, and in extant vertebrates including aquatic salamanders^26^ and the snapping turtle^25^. This evidence in the nighthawk, however, is the first indication of caudal ribs in birds. These structures were not observed in other juveniles in this study (Fig. 3F–H), indicating that more than one mechanism is utilized in birds for vertebral process morphogenesis. The data suggests that extension of the caudal transverse processes and parapophyses themselves occurs via epiphyseal plate-mediated ossification, which is likely a universal avian (if not vertebrate) trait, but further extension can be achieved by incorporating additional bony elements.

Also notable at 7 to 8 weeks old in the central chicken pygostyle is that both halves of the neural arch have not yet fused, and those in turn have not fused to the centrum (Fig. 2D). The initially separate neural arch and centrum components of the vertebral body is a conserved feature in the vertebrate ossification sequence^27^. As development proceeds in the rostral to caudal direction, at any given stage before complete skeletal maturity, the proximal caudal vertebrae will exhibit a greater degree of maturation than at the distal end of the tail, such that the pygostyle region is the least developed. In the chicken, by four years old, bone remodeling results in seamless trabecular bone in all vertebral components (Fig. 2I). Therefore, analogous to humans, ontogenetic changes in ossification sequence in birds cause significant changes in bony vertebral morphology (Fig. 2J,K).

In relation to Cretaceous fossils, these findings indicate that in juveniles, the preserved bony tissue in tail vertebrae differs significantly from adult stages. Assuming the ossification sequence observed in the chicken was conserved in the Mesozoic, caudal vertebrae in young juveniles likely had reduced calcified processes. Also, without fusion of neural arch components to the centra, the overall shape of vertebrae would be elongated compared to later stages. The mineralization state of the vertebral processes notwithstanding, the size and shape of processes on pygostyle vertebrae vary significantly in both extant and Cretaceous birds^28–30^.

**Figure 4:**
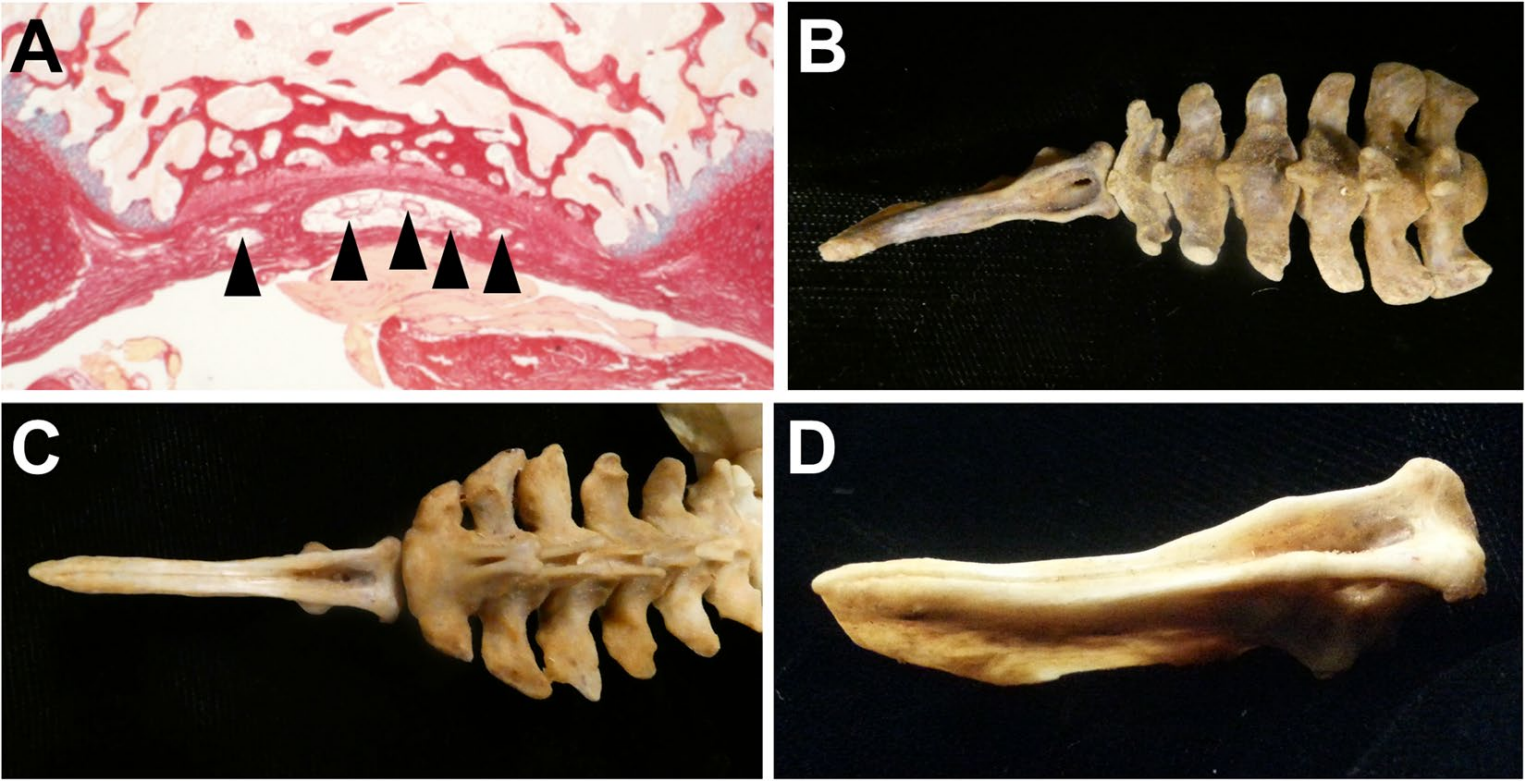
A ventral furrow in caudal vertebrae is a common extant avian trait. A ventral furrow or concave surface in extant avians shelters the ventral tail vasculature. This vasculature includes the branched aorta artery, as well as caudal veins^11^. (**A**) A transverse paraffin section of a 7–8 week old chicken juvenile pygostyle stained with alcian blue and picrosirius red; blood vessels are noted by the black arrowheads. A ventral caudal furrow is evident in the proximal caudal vertebrae in (**B**) a red-tailed hawk, *Buteo jamaicensis* skeletal tail (MOR 1230), especially in the pygostyle region, and in the entire tail in (**C**) a golden eagle, *Aquila chrysaetos* (MOR 116) (ventral views). (**D**) Enlarged ventrolateral view of the golden eagle pygostyle, showing the deeply recessed furrow. MOR is the abbreviation for the Museum of the Rockies.

The ontogenetic morphology changes in caudal vertebrae indicate the recently discovered coelurosaur tail in amber^15^ may require re-evaluation. This specimen consists of a preserved tail with ten vertebrae and attached feathers. Its highly elongated vertebrae and frond-like configuration of rectrices^31^ suggest a long tail, and thus it likely did not belong to the Pygostylia group. However, incompleteness of the material and inferences that the specimen is a juvenile obscure its phylogenetic placement. The specimen was interpreted as non-avian, based on feather morphology, its age (99 Ma), the absence of vertebral processes, and the concave ventral furrow in the vertebrae, not seen in the long-tailed birds *Archaeopteryx* and *Jeholornis*. Concerns regarding the interpretation of the feather morphology have already been raised^32^. In terms of age, fossil beds preserving Cretaceous avian specimens younger than 120 million years old are relatively scarce; there is no reason to exclude the possibility that long-tailed birds existed at that time. In fact, the 75 million-year-old *Rahonavis ostromi* from Madagascar^33^, with a long skeletal tail preserving 13 vertebrae (the length of the distal-most indicating a much greater caudal series), has at times been considered a non-avian dinosaur^34–37^ and at other times a bird^2,33,38^. The lack of vertebral processes in the amber-embedded specimen may be due to its juvenile state, as seen in the unmineralized processes in the juvenile extant bird tails analyzed in this study. Also, a ventral furrow in tail vertebrae is observed in some modern bird tails as a morphological feature housing the tail vasculature (Fig. 4)^22^. These aspects question the categorization of this specimen as non-avian; alternatively, it could have been a long-tailed bird, as has been independently suggested^32^.

### Different avian groups demonstrated varying numbers of pygostyle vertebrae

**Figure 5:**
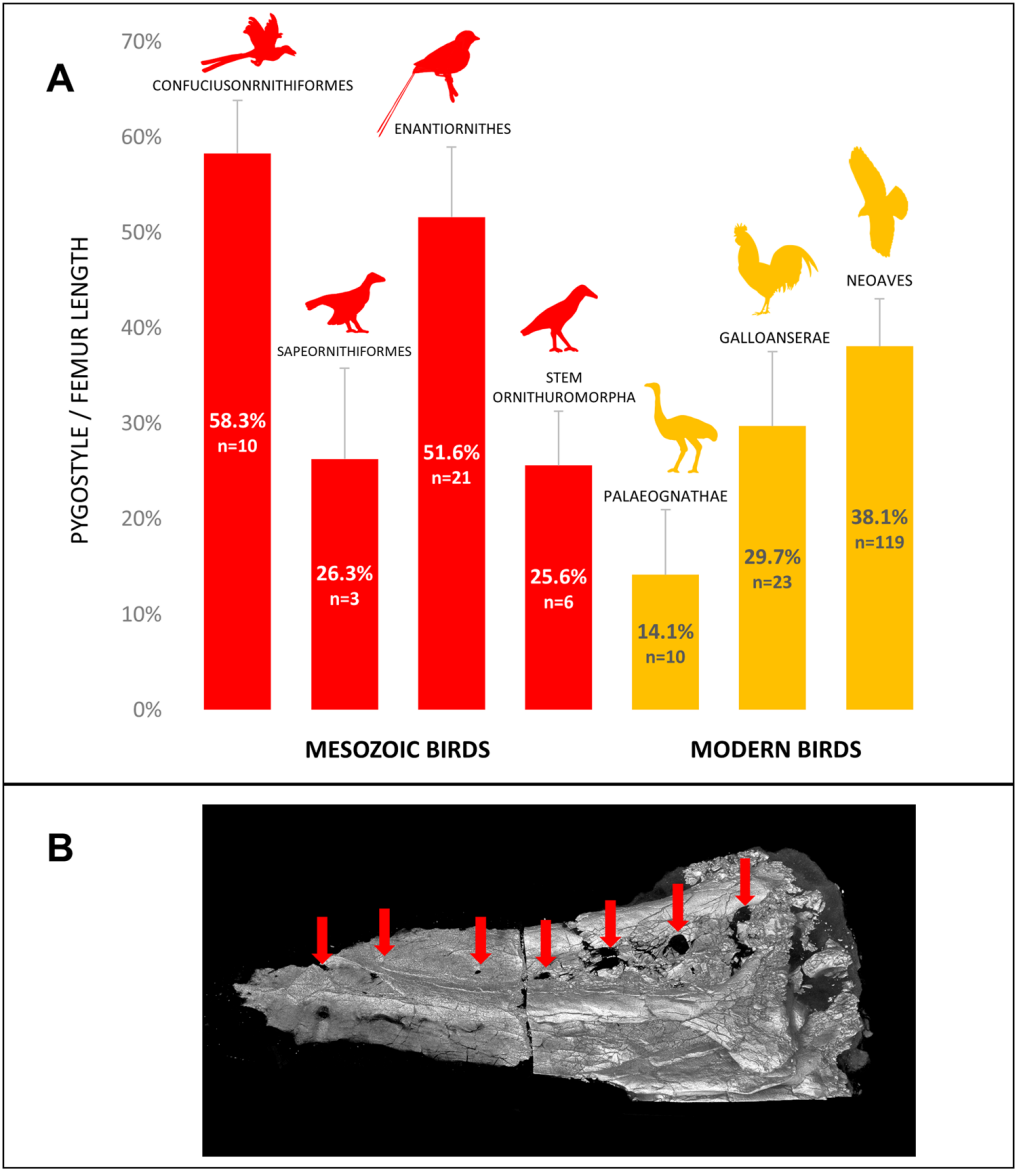
Some Mesozoic birds formed a relatively longer pygostyle than extant birds. (**A**) The percent of adult pygostyle length to femur length was plotted for four different Cretaceous (red) and three different extant (orange) bird groups. The data shows great variation in relative pygostyle lengths overall, and suggests that confuciusornithiformes and enantiornithes incorporated more vertebrae into their pygostyle (n: number of specimens measured). (**B**) MicroCT scan of a *Confuciusornis* pygostyle (NGMC 98-8-2); distal to the left, dorsal to the top. Red arrows indicate intervertebral foramen from incompletely fused neural arches which theoretically correlate to the original number of pygostyle vertebrae before fusion. The break in the middle is due to a thin bone section removed for histology^17^, with the two pygostyle halves digitally reconstructed. Silhouettes were either taken from public domain images on phylopic.org (*Protopteryx*, Matt Martyniuk; *Gallus*, Steven Traver; and *Buteo*, Lauren Anderson), or drawn by D. Rashid (Confuciusornithiformes, Sapeornithiformes, Stem Ornithuromorpha, and Palaeognathae silhouettes). NGMC is the abbreviation for the National Geological Museum of China.

**Figure 6:**
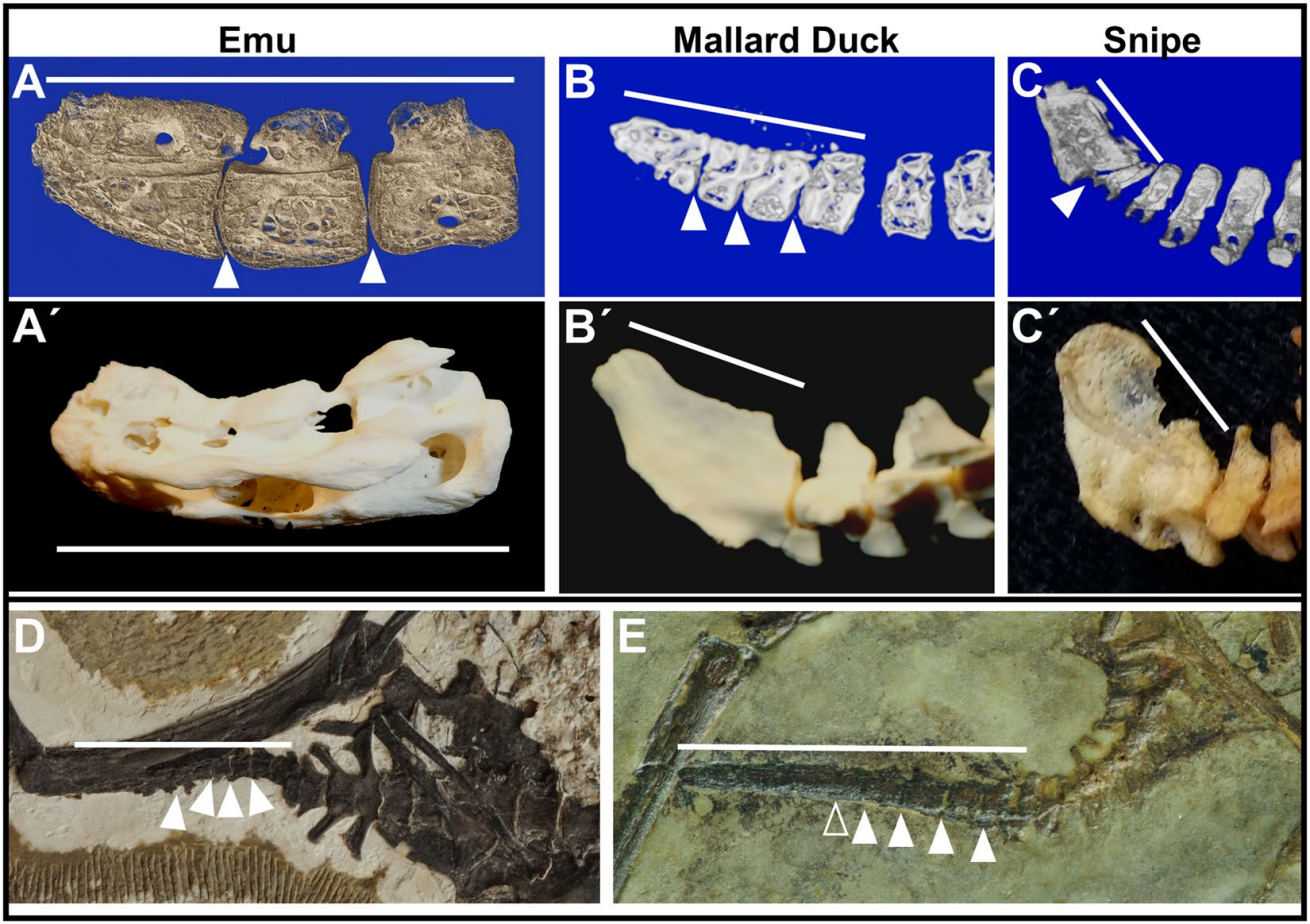
Unfused juvenile pygostyles in extant and Cretaceous birds. White arrows indicate intervertebral spaces (empty arrowhead indicates possible intervertebral space); white bars indicate linear extent of pygostyles; all pygostyles distal to the left, dorsal to the top. (**A**) 5.5 month old emu pygostyle, microCT mid-sagittal view. (**A′**) Adult emu pygostyle. (**B**) Juvenile mallard duck distal tail, microCT mid-sagittal view. (**B′**) Adult mallard duck distal tail. (**C**) Juvenile Wilson’s snipe distal tail, microCT sagittal view. (**C′**) Adult Wilson’s snipe distal tail. (**D**) Juvenile *Archaeorhynchus* IVPP 17075. (**E**) Juvenile enantiornithine, IVPP 15664.

Another complication in interpreting juvenile Cretaceous fossils is the number of vertebrae that were incorporated into pygostyles. To compare the potentially varying numbers of pygostyle vertebrae between extant and Cretaceous birds, pygostyle length as a percent of femur length (an approximation of body size^39^) was determined for a wide range of taxa (Fig. 5A; Supplementary Table 1). The data indicate that the Early Cretaceous confuciusornithids^40^ and enantiornithines^41^ had larger pygostyles relative to body size than other extinct or extant birds. *Sapeornis chaoyangensis*^42^ and the basal ornithuromorphs^2^ included in this analysis, however, had relative pygostyle sizes well within the range of extant birds. Assuming that vertebral length remained approximately constant across clades, a greater number of vertebrae incorporated into the pygostyle was therefore more likely in confuciusornithids and enantiornithines. MicroCT scanning of a *Confuciusornis sanctus* pygostyle (NGMC 98-8-2;^18^) indicates that in this subadult, approximately eight vertebrae ankylosed into the pygostyle, evident by the number of unfused neural arch components (Fig. 3B). A similar estimate is derived from the juvenile enantiornithine specimen IVPP V15664 (Fig. 6E). In the extant birds analyzed in this study, the greatest number of pygostyle vertebrae was five, estimated for the Humboldt penguin (see Supplementary Fig. 1). Applying these combined data to other Cretaceous specimens, the pygostyles in some groups likely incorporated a greater number of vertebrae, and if unfused in juveniles, would further obscure the identification of potential intermediates at the long- to short-tailed transition.

### Pygostyle formation timeframe in multiple bird taxa

As an agricultural animal, the chicken has been highly manipulated, calling for a confirmation of post-hatching pygostyle formation in other avians. Fifteen juvenile bird skins or fixed whole tails, representing thirteen taxa, were analyzed by microCT for the degree of pygostyle fusion relative to corresponding adults. Additionally, juvenile and adult skeletal pygostyle specimens for a fourteenth taxon are included in these analyses (Supplementary Table 2). In all juvenile specimens, with the exception of two or three close to adult size, unfused or partially fused pygostyles were observed (Fig. 6A–C; Supplementary Fig. 1). The three major avian groups, neoaves, galloanseriforms, and paleognaths, were represented in these scans, indicating that post-hatching pygostyle formation is a conserved feature in modern birds, and the same appears to be the case in Mesozoic birds^43^. Consequently, pygostyle fusion is loosely associated with ontogenetic stage, so the widely differing timeframes for fusion in different birds are probably reflected in the timeframes required to achieve skeletal maturity. For example, pygostyle fusion requires five months in the chicken, a 5.5 month old emu has a largely unfused pygostyle, but a California scrub-jay at approximately three weeks old has a completely fused pygostyle (Supplementary Fig. 1). Since neoaves tend to reach adult size much faster, initiate flight earlier, and also tend to be altricial in the developmental mode spectrum^44–47^, a more extensively sampled study would help determine whether pygostyle formation timeframe is better correlated with behavior, developmental mode, or associated genetic group. Among the extant birds analyzed, the trend suggested by this data is that the closer the juvenile to adult size, the greater the chance for complete pygostyle fusion.

### Partial pygostyle fusion in Cretaceous specimens

Since pygostyle fusion in juveniles is a regular feature of extant birds, we sought to determine whether the same phenomenon occurred in Cretaceous avians. Evidence for post hatch pygostyle fusion is found in subadult Sapeornithiformes specimens^28^. In enantiornithines, unfused pygostyle vertebrae in juveniles is evident in IVPP 15564 (Fig. 6E), GMV-2159^48^, CNU VB2005001^49^, and in a juvenile from Spain^50^. Subadult specimens of the basal ornithuromorph *Archaeorhynchus* (IVPP 17075^51^, Fig. 6D) also demonstrate incomplete fusion of the pygostyle. Juvenile confuciusornithiform specimens have not been collected, so pygostyle fusion at younger stages cannot be assessed for this group. Pygostyles differ between subadult and adult confuciusornithids, however; foramina present in subadult pygostyles are lost with ontogeny, indicating remodeling similar to that in neornithines. The total evidence from Cretaceous pygostylians strongly suggests late stage formation of the pygostyle was common, similar to extant birds.

In conclusion, this study reveals several aspects of avian tails that impact the interpretation of Mesozoic specimens. From extant bird tails, we observe that pygostyle formation is a post hatch phenomenon, and vertebral morphology changes dramatically during ontogeny. For *Zhongornis haoae* (and for other juvenile Mesozoic avians), this indicates that lack of a pygostyle does not necessarily indicate an intermediate species in the long- to short-tailed evolutionary transition. Also, the much reduced vertebral processes on juvenile caudal vertebrae can obscure the differentiation between taxa, as seen for the coelurosaur tail in amber. This specimen is reinterpreted here as possibly avian. Enantiornithines and confuciusornithids likely incorporated more vertebrae into their pygostyles, another factor relevant to the transition to short tails. Apart from these findings, tail development includes the generation and subsequent loss of intervertebral discs in the pygostyle region, the formation of vertebrae from disparate types of cartilage, and alternative modes of vertebral process extension. Collectively, these data point to the importance of tail ontogeny in vertebral development and evolutionary analyses.

## Methods

### Animals

#### Extant birds

For histology, Bovan Brown X Rhode Island Red chickens raised at Clemson University Poultry Farm were collected every two weeks after hatching for six months. The 1.5 year old chicken was purchased from a local farm, and the 4+ years old chicken was obtained from Susan Chapman. For the 7 to 8 weeks timepoint, Cornish rock roaster chickens (Springdale Hutterite Colony) were purchased from a local market. Frozen emu carcasses at 3.5 and 5.5 months post-hatch were obtained from the Montana Emu Farm (Kalispell, Montana). For microCT scanning, skin and skeletal specimens were selected from the ornithology collections of the Los Angeles County Museum of Natural History. These specimens, as well as avian skeletal specimens from the Museum of the Rockies, were digitally photographed. For specific specimen numbers, please see the relevant figure legends in both the manuscript proper as well as in the Supplementary Information online. MicroCT scanning was also performed on commercial roaster chickens and on tail tissue extracted from the emu specimens. All harvesting of live animals were conducted in accordance with approved protocols at Clemson University (post-hatch; Animal Use Protocol 2011-041) and at Montana State University (late-stage embryos, IACUC Protocol 2015-26).

#### Fossil specimens

The *Confusiusornis sanctus* pygostyle, in two halves, was prepped away from the rest of the slab-embedded specimen (NGMC 98-8-2), and the base was coated with wax for stabilization prior to microCT scanning. A young enantiornithine (IVPP 15664, taxon unknown) and a juvenile basal ornithuromorph, *Archaeorhynchus* (IVPP 17075, species unknown), were photographed from the Institute of Paleontology and Paleoanthropology (IVPP) collections in China.

### Histology

#### Paraffin embedding and staining

Tail tissue was fixed in either neutral buffered formalin or 4% PFA for two to four days, depending on size. Following one to three days of washing in PBS, the tissue was demineralized by EDTA (Decalcifying Solution EDTA/Sucrose, Newcomer Supply). Demineralized tissue was then processed for wax embedding in a Tissue Tek VIP 6 processor starting with an ethanol series until dehydrated. Tissue was then cleared in Clear Rite followed by Tissue Prep paraffin. Samples were then embedded in Tissue Tek paraffin using the Tissue Tek TEC embedding station. Five to seven micron sections were cut using a Jung RM2035 microtome, and transferred to glass slides. Picrosirius red, which primarily stains collagen fibers, was used as a stain for intervertebral discs, and alcian blue, which stains acidic polysaccharides, was utilized for its general cartilage staining. Masson’s Trichrome stain was utilized to differentiate between mineralized (purple) and unmineralized cartilage (blue). For these stains, paraffin sections were dewaxed with xylenes, and rehydrated through a series of EtOH washes to water. Alcian blue and picrosirius red staining was performed as described in^52^, with minor changes. Briefly, rehydrated sections on slides were stained with alcian blue (0.048% alcian blue dye, 70% EtOH, 20% glacial acetic acid) for 15 minutes, followed by a water wash, then one hour in picrosirius red (0.1% Direct red 80, Sigma, in 1.3% saturated picric acid). The slides were subsequently washed in acidified water (0.5% glacial acetic acid), dehydrated through a series of EtOH washes to xylenes, and mounted in DPX mounting media. For Masson’s Trichrome staining, a modified Masson’s Trichrome method^53^ was performed. Slides were dehydrated with a graded series of ethanol, rinsed in deionized water, stained for 10 min with Mayer’s acid hematoxylin (Sigma MSH-32), rinsed in running distilled water, rinsed in Scott’s tap water, and rinsed again in deionized water. Sections were subsequently stained with Xylidine Ponceau/Acid Fuschin for 2 min (equal volumes of 0.5% xylidine ponceau 2 R CI no. 16150 in 1% acetic acid and 0.5% acid fuchsin CI no. 42685 in 1% acetic acid), rinsed in deionized water, stained for 4 min with 1% phosphomolybdic acid, rinsed in deionized water, stained with light green for 90 seconds (2% light green CI 42095 in 2% citric acid, diluted 1:10 with deionized water prior to use) and rinsed in deionized water. Sections were then dipped two times in 100% Ethanol, cleared in xylene, and finally coverslipped with Permount (Fisher Scientific). Higher magnification images were obtained with a Zeiss Axioscope A.1 in conjunction with a Jenoptik ProgRes C14 Plus digital camera and accompanying software. Lower magnification images of whole sections were obtained using a Canon CanoScan 9000 F Mark II scanner with slide holder.

#### Wholemount and von Kossa staining

To differentiate mineralized bone from unmineralized cartilage, alcian blue and alizarin red staining was performed in wholemount tissue, and von Kossa was performed on unfixed, sliced tissue. Alizarin red is a calcium-sensitive dye that stains mineralized tissue red, and von Kossa is a silver-based stain that is phosphate sensitive, and stains mineralized tissue black. Alcian blue and alizarin red staining was performed using a protocol modified from^54^. Briefly, tissue was fixed in 95% EtOH for five days, followed by co-staining with alcian blue and alizarin red (0.015% alcian blue, 0.005% alizarin red-S, 5% glacial acetic acid, and 71.25% EtOH) for 4 days at 40 °C. Tissue was then cleared in 2% KOH for 3 days, followed by clearing in 1% KOH in 20% glycerol until internal structures were visible. Final clearing was achieved by gentle rocking in 50%, 80%, then 100% glycerol for 2 days each. For von Kossa staining, a juvenile chicken pygostyle dissected free of skin and surrounding tissue was sliced by hand in transverse sections (approx. 1.5 mm thick) with a razor blade. The sections were transferred to a concave glass slide, and von Kossa stained according to the kit instructions (Silver staining kit acc. to von Kossa; EMD Millipore). The reaction was allowed to proceed under bright incandescent light for 5 minutes for the silver nitrate solution, followed by 5 minutes in the sodium thiosulfate solution. Stained tissue slices were subsequently imaged with either a Unitron TCS tablet mounted on a Zeiss Stemi 200c dissecting microscope for lower resolution images, or a Zeiss Stemi SV11 stereoscope with a Jenoptik ProgRes C14 camera using associated software for higher resolution imaging. All staining procedures complied with mandated Montana State University biosafety regulations.

### Pygostyle and femur length measurements

To investigate how pygostyle relative size evolved throughout the avian lineage, the mean pygostyle length/femur length was calculated for the seven extinct and extant clades; note that stem Ornithuromorpha is paraphyletic. The greatest dimension of pygostyle lengths were measured from the anterior-most ventral process to the distal tip of the lamina. This is a conservative measurement for estimating relative vertebral count in the pygostyle, considering some adult pygostyles are elaborated dorsoposteriorly, making pygostyles appear potentially larger relative to the number of vertebrae incorporated into them. Total femur length was measured as a proxy for body size^39^. For extant taxa, total pygostyle length and total femur length of 152 adult extant specimens (64 representative species from major extant clades) were measured from the Los Angeles County Natural History Museum (LACM) and Museum of the Rockies (MOR) ornithology collections. Whenever possible (40 of the 64 species), at least three specimens per species were measured. Next, the mean pygostyle length/femur length was calculated for the three major extant clades. For extinct taxa, the pygostyle/femur lengths of 40 specimens were collected from published sources^28^. Osteological specimens and images from^55^ were measured with the appropriate calipers, and measurements from Wang and colleagues were provided in their article (Table 1 in^28^). The mean pygostyle length/femur length for the seven extinct and extant clades was subsequently calculated and plotted by bar graph. The upper 99% Confidence interval was calculated for each clade, noted in Supplementary Table 2. For the bar graph, silhouettes were either taken from public domain images on phylopic.org (*Protopteryx*, Matt Martyniuk; *Gallus*, Steven Traver; and *Buteo*, Lauren Anderson), or drawn by D. Rashid (Confuciusornithiformes, Sapeornithiformes, Stem Ornithuromorpha, and Palaeognathae silhouettes).

### 3D imaging by microCT analysis

MicroCT of formalin-fixed chicken and emu tissue was performed by the Bone Pathology Laboratory at the Mayo Clinic (Rochester, MN), and the bird skins and skeletal microCT scans (including the *Confuciusornis sanctus* pygostyle) were performed by the Molecular Imaging Center at the University of Southern California (Los Angeles, CA). The CT scans were performed in air on a XT H 225 S T micro-CT scanner (Nikon Metrology, Brighton, MI) with a PerkinElmer 1621 detector at 70 kVp, 100 uA, 1000 ms exposure, 1000 projections/360 degree and 24 dB gain to create an isotropic 10–16 micron voxel volume of each specimen. Raw data was reconstructed in CT Pro 3D v 4.3.4 (XT Software Suite, Nikon Metrology) and volume rendering was performed using Amira® 5.3.3 (Visualization Sciences Group, Burlington, MA). For the bird skin specimens, extraneous surrounding tissue was pared away digitally, to reveal tail vertebrae anatomy. Various views of specimen tail vertebrae were generated, including dorsal and sagittal surface views, as well as mid-sagittal plane views.

### Statistics

Statistical analyses for this study are limited to data presented in Fig. 5 and in Supplementary Table 1. The bar graph in Fig. 5 shows the mean total pygostyle length/femur length of specimens within each bird group with indicated error bars (the bar graph and the error bars were created using Microsoft Excel). The error bars represent the upper 99% CI. The value of n (sample size) represents the number of specimens measured, noted in SupplementaryTable 1.

### Availability of Materials and Data

With the exception of m*i*croCT raw data, all data generated or analyzed during this study are included in this published article (and its Supplementary Information files). MicroCT raw data is available upon request from D. Rashid. All museum specimens described are available upon request, on site, from their respective museums.

## Electronic supplementary material

Supplementary Information

Supplementary Table 1
