# Supplementary material for: Avian tail ontogeny, pygostyle formation, and interpretation of juvenile Mesozoic specimens
Source: Sci Rep. 2018 Jun 13;8:9014. doi: 10.1038/s41598-018-27336-x (PMC5997987; doi:10.1038/s41598-018-27336-x)
Supplement: Supplementary file 1 — Supplementary Information [file 41598_2018_27336_MOESM1_ESM.pdf]

Supplementary Information

Avian tail ontogeny, pygostyle formation, and interpretation of juvenile Mesozoic specimens

Authors: Dana J. Rashid, Kevin Surya, Luis M. Chiappe, Nathan Carroll, Kimball L. Garrett, Bino Varghese, Alida Bailleul, Jingmai K. O'Connor, Susan C. Chapman, and John R. Horner

Supplemental data for this study includes two tables and one figure. Note that Supplementary Table 1 is a separate excel file with multiple tabs.

Supplementary Figure 1. Fusion of pygostyle vertebrae occurs as a post-hatching event across extant bird clades. MicroCT (in greyscale) and digital photographs (in color) of juvenile and adult distal tails were compared for their relative degree of pygostyle fusion. All tails are distal to the left, proximal to the right, and not to scale. White arrowheads indicate unfused pygostyle vertebral junctions; open arrowheads indicate possible unfused pygostyle vertebral junctions, and white bars indicate the extent of the pygostyle region. The proximal boundaries of the juvenile pygostyles are estimated from adult dimensions and morphological features. **A,** paleognathous birds; **Aa-Ac,** emu *Dromaius novaehollandiae*, surface sagittal views; **Aa,** 3.5 month old; **Ab,** 5.5 month old; **Ac,** adult, MOR 186; **Ad-Af,** elegant crested tinamou, *Eudromia elegans;* **Ad,** juvenile, surface sagittal view, LACM 85562; **Ae,** same specimen as in Ad, mid-sagittal view, yellow arrow indicates spinal cord channel; **Af,** adult, surface sagittal view, LACM 116227. **B,** galloanseriform birds, surface sagittal views; **Ba-Ba´,** mallard duck *Anas platyrhynchos;* **Ba,** juvenile, LACM 110758; **Ba´,** adult, MOR 1158; **Bb-Bb´,** domestic chicken *Gallus gallus*; **Bb,** 7-8 week old juvenile; **Bb´,** 1.5 year old adult; **Bc-Bc´,** bush quail, *Perdicula asiatica*; **Bc,** juvenile, LACM 85646; **Bc**´, adult, LACM 90214. **C,** neoaves; **Ca- Ca´,** California scrub jay, *Aphelocoma californica*; **Ca,** juvenile, LACM 111600; **Ca**´, adult, LACM 119559; **Cb-Cb´,** least sandpiper, *Calidris minutilla*; **Cb,** juvenile LACM 80368; **Cb´,** adult, LACM 113110; **Cc-Cc´,** lesser nighthawk, *Chordeiles acutipennis;* **Cc,** juvenile, LACM 11218; **Cc´,** adult, LACM 73857; **Cd-Cd´,** sunbittern, *Eurypyga helias*; **Cd,** juvenile LACM 104451; **Cd´,** adult, LACM 90009; **Ce-Ce´,** Wilson's snipe, *Gallinago delicata*; **Ce,** juvenile, LACM 80241; **Ce´,** adult, LACM 113137; **Cf-Cf´,** western screech owl, *Megascops kennicotti*; **Cf,** juvenile LACM 111461; **Cf´,** adult LACM 105612; **Cg-Cg´,** red-headed woodpecker, *Melanerpes erythrocephalus*; **Cg,** juvenile LACM 6313; **Cg´,** adult LACM 118444; **Ch-Ch´,** Humboldt penguin, *Spheniscus humboldti*; **Ch,** juvenile LACM 888834; **Ch´,** adult LACM 116234; **Ci-Ci´´,** elegant tern, *Thalasseus elegans*; **Ci,** young chick, the caudal vertebrae are still in the process of ossifying, C is centrum and NA is neural arch, LACM 109304; **Ci´,** fledgling near adult size, LACM 116139; **Ci´´,** adult LACM 117720.

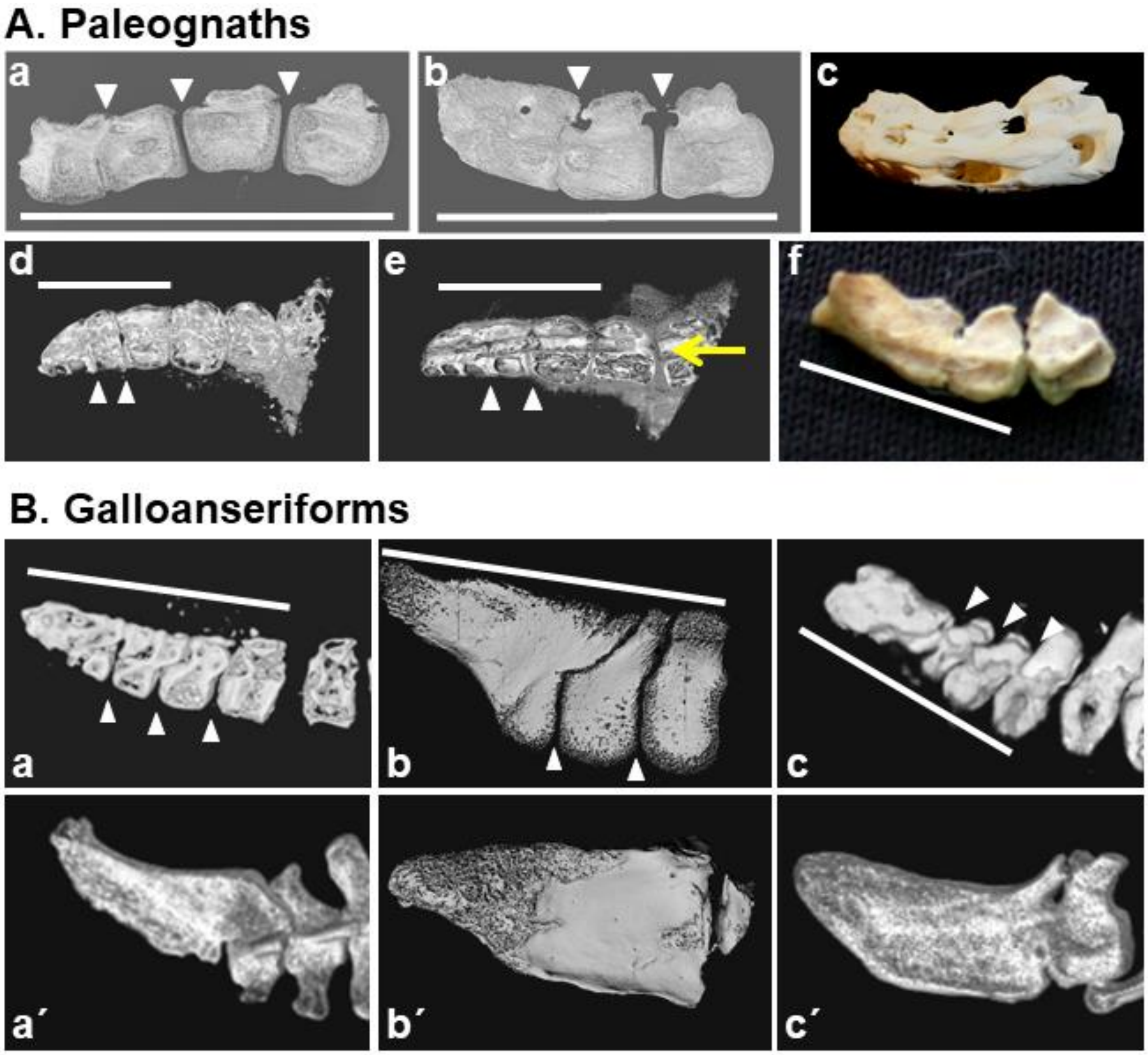
A. Paleognaths
a
b
c
d
e
f
B. Galloanseriforms
a
b
c
a′
b′
c′

C. Neoaves

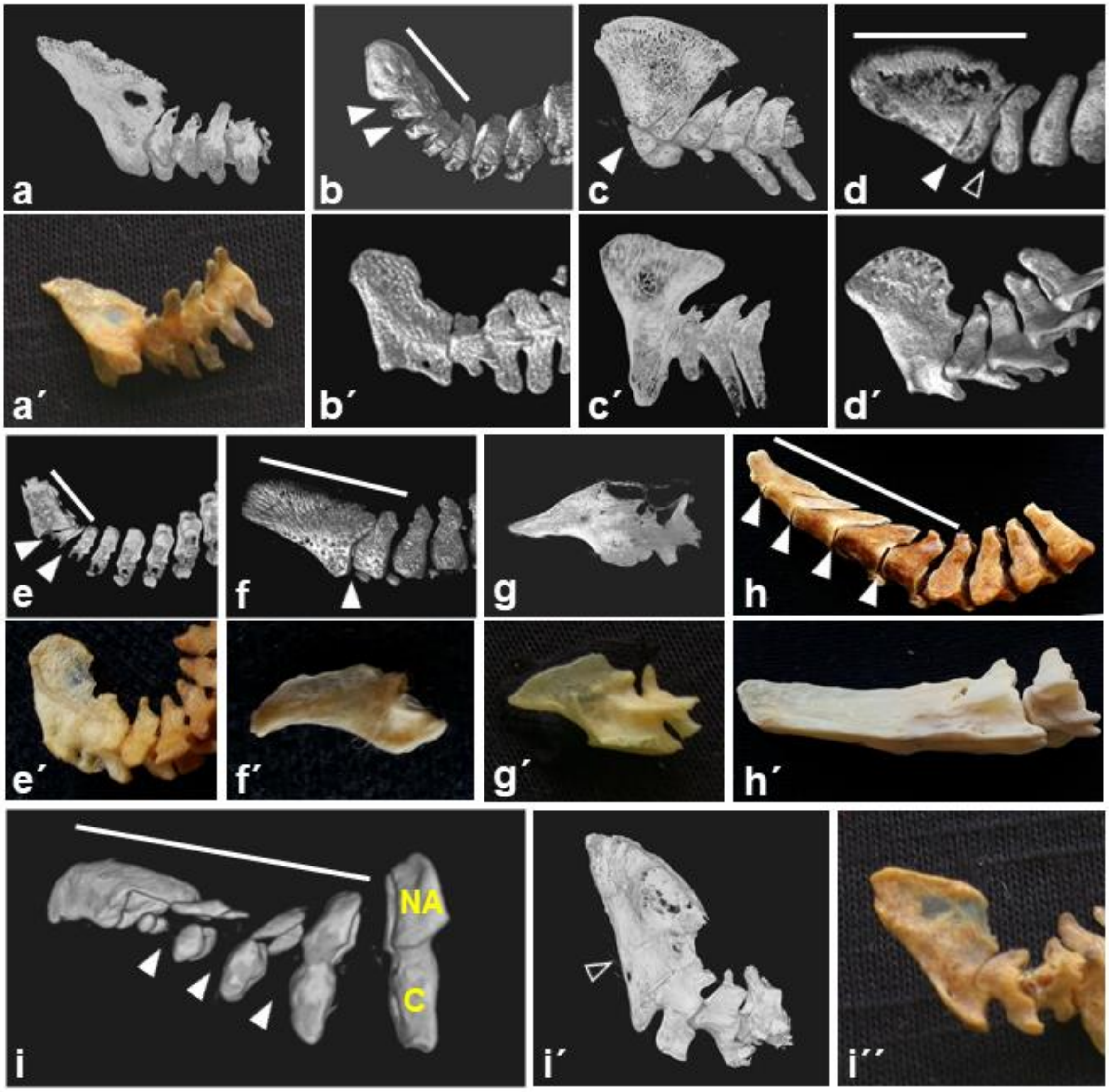

Supplementary Table 2. **Assessed pygostyle fusion and relative body size for the extant avian specimens analyzed (referred to in *Pygostyle formation timeframe in multiple bird taxa)*.** Relative ontogenetic stages of µCT-scanned immature bird specimens (top), skeletal specimen (middle) and histology-stained tissue specimens (bottom) and whether their pygostyles have completely fused. Fusion appears to occur medially to distally, so for some specimens, surface views were required, where for others, mid-sagittal views were sufficient. For LACM (Natural History Museum of Los Angeles County) skin specimens, tarsometatarsus length was used because it was the only outwardly measurable limb element. Developmental mode data follow [1] and was inferred based on [1, 2] and descriptions in [3] when data were unavailable. Skeletal maturity data follow [4] for *Dromaius novaehollandiae* and [5] for *Gallus gallus* (white leghorn).

LACM CODE	TAXON	COMMON NAME	CLADE	TARSO- -METATARSUS LENGTH (mm)	MEAN ADULT TARSO- -METATARSUS LENGTH (mm)	% ADULT BODY SIZE	COMPLETE FUSION?	DEVELOPMENTAL MODE
110758	*Anas platyrhynchos*	Mallard	Galloanserae	19.9	48.4	41.1%	No	Precocial
111600	*Aphelocoma californica*	California scrub-jay	Neoaves	37.2	40.9	91.0%	Yes	Super altricial
80368	*Calidris minutilla*	Least sandpiper	Neoaves	17.7	18.7	94.7%	No	Precocial
111218	*Chordeiles acutipennis*	Lesser nighthawk	Neoaves	13.6	14.3	95.1%	No	Semi altricial
85562	*Eudromia elegans*	Elegant crested-tinamou	Palaeognathae	31.9	48.3	66.0%	No	Precocial
104451	*Eurypyga helias*	Sunbittern	Neoaves	29.1	50.1	58.1%	No	Semi precocial
80241	*Gallinago gallinago*	Common snipe	Neoaves	19.4	32.0	60.6%	No	Precocial
111461	*Megascops kennicottii*	Western screech owl	Neoaves	26.5	34.6	76.6%	No	Altricial
6313	*Melanerpes erythrocephalus*	Red-headed woodpecker	Neoaves	25.4	23.5	108.1%	Yes	Altricial
85647	*Perdicula asiatica*	Jungle bush-quail	Galloanserae	8.0	25.6	31.3%	No	Precocial
109304	*Thalasseus elegans*	Elegant tern	Neoaves	17.6	29.1	60.5%	No	Precocial
116139	*Thalasseus elegans*	Elegant tern	Neoaves	27.7	29.1	95.2%	Possibly	Precocial
LACM CODE	TAXON	COMMON NAME	CLADE	FEMUR LENGTH (mm)	MEAN ADULT FEMUR LENGTH (mm)	% ADULT BODY SIZE	COMPLETE FUSION?	DEVELOPMENTAL MODE
888834	*Spheniscus humboldti*	Humboldt penguin	Neoaves	83.3	82.2	101.3%	No	Altricial
	TAXON	COMMON NAME	CLADE	AGE (days)	AGE AT SKELETAL MATURITY (days)	% AGE AT SKELETAL MATURITY	COMPLETE FUSION?	DEVELOPMENTAL MODE
	Dromaius novaehollandiae	Emu	Palaeognathae	105	365	28.8%	No	Precocial
	Dromaius novaehollandiae	Emu	Palaeognathae	165	365	45.2%	No	Precocial
	Gallus gallus	Domestic chicken	Galloanserae	8	140	5.7%	No	Precocial
	Gallus gallus	Domestic chicken	Galloanserae	58	140	41.4%	No	Precocial
	Gallus gallus	Domestic chicken	Galloanserae	64	140	45.7%	No	Precocial
	Gallus gallus	Domestic chicken	Galloanserae	88	140	62.9%	No	Precocial
	Gallus gallus	Domestic chicken	Galloanserae	105	140	75.0%	No	Precocial
	Gallus gallus	Domestic chicken	Galloanserae	126	140	90.0%	No	Precocial
	Gallus gallus	Domestic chicken	Galloanserae	168	140	120.0%	Yes	Precocial

1. Botelho, J.F., and Faunes, M. (2015). The evolution of developmental modes in the new avian phylogenetic tree. Evolution & Development *17*, 221-223.
2. Rodewald, P. (2015). The Birds of North America. (Cornell Laboratory of Ornithology).
3. Dial, K.P. (2003). Evolution of avian locomotion: correlates of flight style, locomotor modules, nesting biology, body size, development, and the origin of flapping flight. The Auk *120*, 941-952.
4. Regnault, S., Pitsillides, A.A., and Hutchinson, J.R. (2014). Structure, ontogeny and evolution of the patellar tendon in emus (*Dromaius novaehollandiae*) and other palaeognath birds. PeerJ *2*, e711.
5. Church, L.E., and Johnson, L.C. (1964). Growth of long bones in the chicken. Rates of growth in length and diameter of the humerus, tibia, and metatarsus. American Journal of Anatomy *114*, 521-538.
